# A universal in silico V(D)J recombination strategy for developing humanized monoclonal antibodies

**DOI:** 10.1186/s12951-022-01259-2

**Published:** 2022-01-31

**Authors:** Yuan-Chin Hsieh, Jun-min Liao, Kuo-Hsiang Chuang, Kai-Wen Ho, Shih-Ting Hong, Hui-Ju Liu, Bo-Cheng Huang, I-Ju Chen, Yen-Ling Liu, Jaw-Yuan Wang, Hsiang-Lin Tsai, Yu-Cheng Su, Yen-Tseng Wang, Tian-Lu Cheng

**Affiliations:** 1 Center for Biomarkers and Biotech Drugs, Kaohsiung Medical University, 100 Shih-Chuan First Road, Kaohsiung, 80708 Taiwan; 2 Graduate Institute of Pharmacognosy, Taipei Medical University, 250 Wuxing Street, Taipei, 11031 Taiwan; 3 Ph.D. Program for Clinical Drug Discovery From Botanical Herbs, Taipei Medical University, 250 Wuxing Street, Taipei, 11031 Taiwan; 4 Graduate Institute of Medicine, College of Medicine, Kaohsiung Medical University, 100 Shih-Chuan First Road, Kaohsiung, 80708 Taiwan; 5 Institute of Biomedical Sciences, National Sun Yat-Sen University, 70 Lien-hai Road, Kaohsiung, 804 Taiwan; 6 School of Medicine for International Students, I-Shou University, No.8, Yida Rd., Jiaosu Village Yanchao District, Kaohsiung, 82445 Taiwan; 7 School of Medicine, I-Shou University, No.8, Yida Rd., Jiaosu Village Yanchao District, Kaohsiung, 82445 Taiwan; 8 Department of Biomedical Science and Environmental Biology, Kaohsiung Medical University, 100 Shih-Chuan First Road, Kaohsiung, 80708 Taiwan; 9 Division of Colorectal Surgery, Department of Surgery, Kaohsiung Medical University Hospital, Kaohsiung Medical University, 100 Shih-Chuan First Road, Kaohsiung, 80708 Taiwan; 10 Department of Surgery, Faculty of Medicine, Kaohsiung Medical University, 100 Shih-Chuan First Road, Kaohsiung, 80708 Taiwan; 11 Graduate Institute of Clinical Medicine, College of Medicine, Kaohsiung Medical University, No.100, Tzyou 1st Rd., Sanmin Dist., Kaohsiung, 80756 Taiwan; 12 Center for Cancer Research, Kaohsiung Medical University, 100 Shih-Chuan First Road, Kaohsiung, 80708 Taiwan; 13 Department of Biological Science and Technology, National Yang Ming Chiao Tung University, No. 1001, Daxue Rd. East Dist., Hsin-Chu, 300 Taiwan; 14 School of Post-Baccalaureate Medicine, College of Medicine, Kaohsiung Medical University, 100 Shih-Chuan First Road, Kaohsiung, 80708 Taiwan

**Keywords:** Antibody, Humanized antibody, TNF-α, Root mean squared deviation (RMSD), Molecular dynamics

## Abstract

**Background:**

Humanization of mouse monoclonal antibodies (mAbs) is crucial for reducing their immunogenicity in humans. However, humanized mAbs often lose their binding affinities. Therefore, an in silico humanization method that can prevent the loss of the binding affinity of mAbs is needed.

**Methods:**

We developed an in silico V(D)J recombination platform in which we used V(D)J human germline gene sequences to design five humanized candidates of anti-tumor necrosis factor (TNF)-α mAbs (C1–C5) by using different human germline templates. The candidates were subjected to molecular dynamics simulation. In addition, the structural similarities of their complementarity-determining regions (CDRs) to those of original mouse mAbs were estimated to derive the weighted interatomic root mean squared deviation (wRMSD_i_) value. Subsequently, the correlation of the derived wRMSDi value with the half maximal effective concentration (EC50) and the binding affinity (K_D_) of the humanized anti-TNF-α candidates was examined. To confirm whether our in silico estimation method can be used for other humanized mAbs, we tested our method using the anti-epidermal growth factor receptor (EGFR) a4.6.1, anti-glypican-3 (GPC3) YP9.1 and anti-α4β1 integrin HP1/2L mAbs.

**Results:**

The R^2^ value for the correlation between the wRMSD_i_ and log(EC50) of the recombinant Remicade and those of the humanized anti-TNF-α candidates was 0.901, and the R^2^ value for the correlation between wRMSD_i_ and log(K_D_) was 0.9921. The results indicated that our in silico V(D)J recombination platform could predict the binding affinity of humanized candidates and successfully identify the high-affinity humanized anti-TNF-α antibody (Ab) C1 with a binding affinity similar to that of the parental chimeric mAb (5.13 × 10^−10^). For the anti-EGFR a4.6.1, anti-GPC3 YP9.1, and anti-α4β1 integrin HP1/2L mAbs, the wRMSD_i_ and log(EC50) exhibited strong correlations (R^2^ = 0.9908, 0.9999, and 0.8907, respectively).

**Conclusions:**

Our in silico V(D)J recombination platform can facilitate the development of humanized mAbs with low immunogenicity and high binding affinities. This platform can directly transform numerous mAbs with therapeutic potential to humanized or even human therapeutic Abs for clinical use.

**Graphical Abstract:**

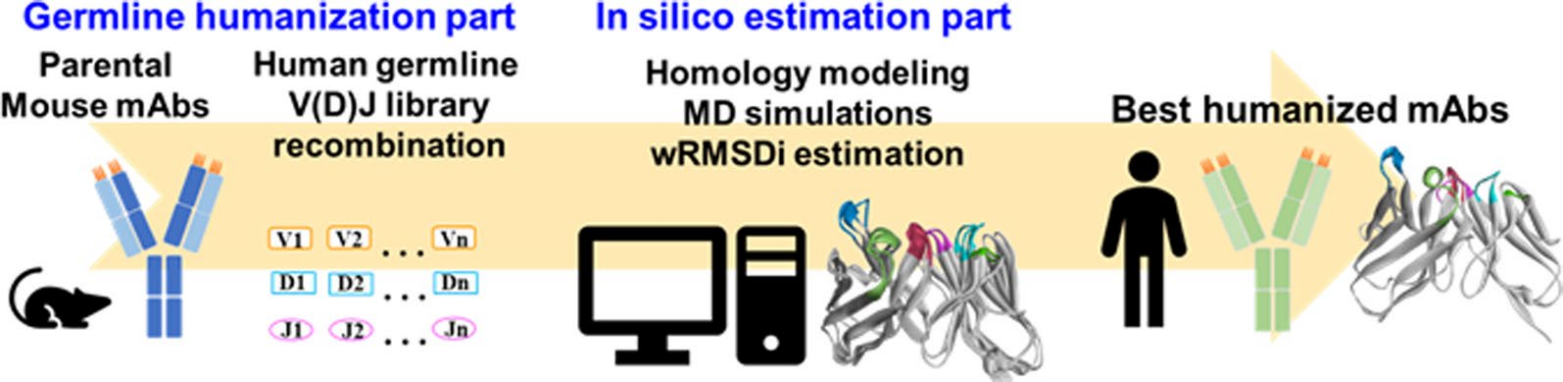

**Supplementary Information:**

The online version contains supplementary material available at 10.1186/s12951-022-01259-2.

## Introduction

Therapeutic monoclonal antibodies (mAbs) have been approved for the treatment of various human diseases including cancer, infections, and immune disorders [1]. The global mAb market is predicted to reach US$179.56 billion by 2025, with a compounded annual growth rate of 11.9% [2]. The conventional method used to develop effective mAbs involves the immunization of mice with the target antigen (Ag) and the generation of a hybridoma to acquire the Ag-specific antibody (Ab) [3]. Mouse mAbs cannot be directly applied to humans because of the immunogenicity of the human anti-mouse Ab (HAMA) in humans; therefore, chimeric mAbs fusing variable regions with human constant region domains were developed. Furthermore, to reduce immunogenicity, humanized mAbs integrating human frameworks were developed. Hwang et al. reported that the anti-Ab responses of mouse, chimeric, and humanized Abs were 84%, 40%, and 9%, respectively [4]. The humanization of murine mAbs can reduce their immunogenicity in humans. Thus, a reliable approach for humanizing potential mouse mAbs is necessary for developing therapeutic mAbs.

Approaches currently used to humanize mAbs often lead to the loss of binding affinities [5]. The conventional humanization approach involves grafting complementarity-determining regions (CDRs) into a suitable human template. For example, Kettleborough et al. grafted the CDR of a mouse anti-epidermal growth factor (EGFR) mAb (mAb-425) into a human template and found no detectable signal until some framework residues of humanized mAbs were mutated back to those of mice; this process is termed as back mutation [6]. Cristina et al. found that the humanized mouse anti-EGFR mAb did not inhibit EGFR in the grafted version; however, the variant with back mutations inhibited EGFR [7]. In addition, some humanized mAbs still exhibited immunogenicity [8]. Thus, a more favorable method for selecting the human mAb template is required. The germline humanization approach involves the grafting of the CDRs of a mouse mAb into a human Ab germline gene sequence with the highest similarity [9, 10]. Because of the low intraclonal somatic hypermutation of human germline genes, grafted mAbs might exhbit low immunogenicity [11]. Using the germline humanization approach, Tan et al. aligned the V region of the murine antihuman CD28 mAb to the human germline gene sequence. They demonstrated that the Ag-binding affinities of chimeric and humanized Abs were 20 and 630 nM and indicated moderate loss of binding affinity [12]. Pelat et al. humanized 35PA_83_ with human germline sequences and regained affinity through additional back mutations [13]. These findings indicated the importance of back mutations in maintaining the binding affinity of mAbs. However, selecting human mAb templates and the subsequent back mutations requires numerous tests, which can be time consuming and expensive. Therefore, an in silico method for predicting humanized candidates with a high binding affinity should be developed.

The binding affinities of humanized candidates and mouse mAbs can be estimated by determining the binding energies of Ab–Ag complexes in all-atom simulations [14]. However, the cocrystal structures of mouse mAbs were typically not resolved when they were not humanized. Only few in silico mAb humanization methods have been used in the absence of Ab–Ag crystal structures. For example, Pier et al. developed TabHu, a sequence-based web server for Ab humanization that can be used to calculate the contact probabilities of mAbs, and employed it to humanize the anti-EGFR Ab a4.6.1 [15]. However, the lack of structural information would still lead to lower binding affinities. Bujotzek et al. used a learning-based method to determine the VH–VL orientation and selected similar human template orientations to humanize the CD81K04 and CD81K13 mAbs [16] However, this method cannot be used to determine the binding affinity of humanized candidates derived using the same human template. A comparison of CDRs between mouse mAbs and their humanized candidates would be a more effective method to estimate the binding abilities of humanized candidates in the absence of Ab–Ag cocrystal structures. However, the structural information of mAbs and their humanized variants must be determined using homology modeling methods that require high sequence similarities [17–20]. Molecular dynamics (MD) simulation can be used with homology modeling to improve the accurate structure of mAbs [21–23]. To compare CDRs and protein structures, interatomic root mean squared deviation (RMSD_i_) has been widely used [24–27]. Weights can be added to RMSD_i_ to compare flexible proteins and limit the comparison to certain regions; hence, this approach would be suitable for comparing the CDRs of humanized candidates with those of parental mouse mAbs [28]. Therefore, the use of a method involving MD simulation to improve the homology modeling of mouse mAbs and their humanized candidates and weighted RMSD_i_ (wRMSD_i_) to compare the structural ensembles of CDRs can be a rigorous and easy in silico method for evaluating the binding affinities of humanized and mouse mAbs in the absence of Ab–Ag cocrystal structures.

**Fig. 1:**
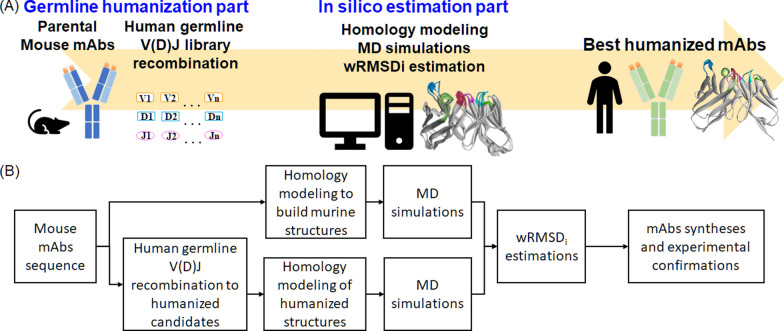
**A** Flowchart and **B** schematic diagram of the in silico V(D)J recombination platform

Herein, we propose an in silico V(D)J recombination platform consisting of two components: (1) In the human germline V(D)J recombination component, the variable regions of the heavy chain (VH) and light chain (VL) of murine Abs were aligned to the human V(D)J region. The human germline sequences with the highest similarities were selected as frameworks for V(D)J recombination and then back mutated to generate many germline humanized candidates. (2) In the in silico mAb estimation component, murine and humanized mAbs were subjected to homology modeling and MD simulation. The resulting trajectories were analyzed to derive RMSD_i_ by comparing the CDRs of murine and humanized candidates. Furthermore, the RMSD_i_ was weighted by referencing the solvent accessible surface area (SASA) and dynamic fluctuations in CDR residues to reduce the effect of nonbinding CDR residues or large vibration motions. The resulting wRMSD_i_ was used to predict the affinities of humanized mAb candidates (Fig. 1). Using the platform, we generated five humanized candidates of anti-tumor necrosis factor (TNF)-α mAb: C1–C5. The candidates were subjected to MD simulation and compared with the parental mouse mAb to derive the wRMSD_i_ to estimate their binding affinities. The wRMSD_i_ was correlated with the half maximal effective concentration (EC50) of the candidates by performing a direct enzyme-linked immunosorbent assay (ELISA). To confirm the predicted binding affinities, we examined the correlation of the wRMSD_i_ with the K_D_ of the candidates. Furthermore, to evaluate whether our in silico estimation method can be used for other humanized mAbs, we tested our method using anti-EGFR [29], anti-GPC3 [30], and anti-α4β1 integrin [31] mAbs (Supplementary information).

In the human germline V(D)J recombination component, the parental mouse mAb sequence was matched with the human germline gene sequences. Subsequently, V(D)J recombination was performed to generate humanized candidates. In the in silico estimation component, the humanized candidates were subjected to homology modeling and MD simulation. The trajectories of the humanized candidates were compared with those of the parental mouse mAb to determine the wRMSD_i_ and the most favorable humanized mAb candidates. The candidates were synthesized and examined in experiments.

## Results

### In silico V(D)J recombination of humanized anti-TNF-α mAbs

**Fig. 2:**
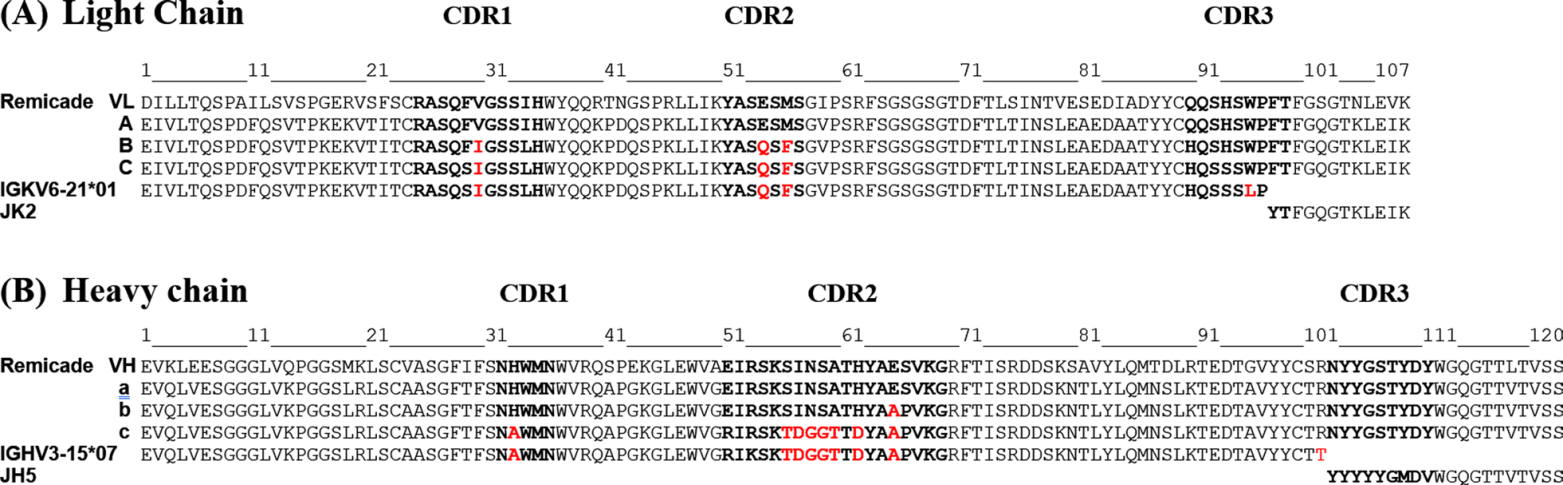
Sequence alignment of the light (**A**) and heavy (**B**) chain of humanized anti-TNF-α Abs and the human germline genes. The CDR regions (bold) are defined by Kabat and conserved during the humanization process to increase the diversity of sequences; some key residues were replaced by the germline template (red)

We used our human germline V(D)J recombination platform to humanize anti-TNF-α mAbs (C1–C5). The amino acid sequences of the chimeric Ab (Remicade) were aligned to the human germline sequences [32]. IGKV6-21*01-JK2 and IGHV3-15*07-JH5 were selected as the human immunoglobulin (lg) frameworks for CDR grafting for the VL and VH, respectively. Thus, we designed three VL (A, B, and C) and VH (a, b, c) grafting conditions. The detailed sequences are presented in Fig. 2. To increase the feasibility of the calculations, nine humanized candidates were generated by pairing the VL (A, B, C) and VH (a, b, c). All the nine humanized anti-TNF-α mAb candidates were subjected to MD simulation, and the resulting trajectories were compared with those of Remicade. Their wRMSD_i_ was derived by comparing interatomic distances between different trajectories and was adjusted with weights for accessibility and movability. The binding affinities of the nine mAbs were in the following order: C1 (A + a) > C2 (A + b) > (B + c) > C3 (B + b) > C4 (B + a) > (A + c) > (C + c) > (C + a) > C5 (C + b). The estimated wRMSD_i_ are listed in Table 1. We observed that the humanized candidates can be divided into three groups: those with the highest structural similarity, namely C1 (A + a), C2 (A + b), and B + c; those with a moderate structural similarity, namely C3 (B + b), C4 (B + a), A + c, and C + c; and those with the lowest structural similarity, namely C + a and C5(C + b). Therefore, we selected C1 and C2, C3 and C4, and C5 from the groups with the highest, moderate, and lowest structural similarity, respectively, for further protein synthesis and functional tests. The measured wRMSD_i_ were 1.207, 1.214, 1.327, 1.400, and 1.603 for the humanized anti-TNF-α candidates C1, C2, C3, C4, and C5, respectively, and 1.137 for the recombinant Remicade mAb (Table 1). If a humanized variant had a smaller wRMSD_i_ than did the mouse mAb, then the binding affinity of the variant to Ags was similar to that of the mouse mAb. The predicted order of the binding affinities was as follows: C1 > C2 > C3 > C4 > C5.

**Table 1:** The wRMSD_i_ of our Remicade humanized candidates and their similarity rankings

Candidates	wRMSD_i_ (Å)	Similarity ranking	EC50 (ng/mL)
C1 (A + a)	1.207	1	25.83
C2 (A + b)	1.214	2	26.08
B + c	1.308	3	–
C3 (B + b)	1.327	4	513.65
C4 (B + a)	1.400	5	545.70
A + c	1.452	6	–
C + c	1.453	7	–
C + a	1.465	8	–
C5 (C + b)	1.469	9	> 1000
rRemicade	1.137	–	19.03

### In vitro binding of humanized anti-TNF-α mAb candidates

**Fig. 3:**
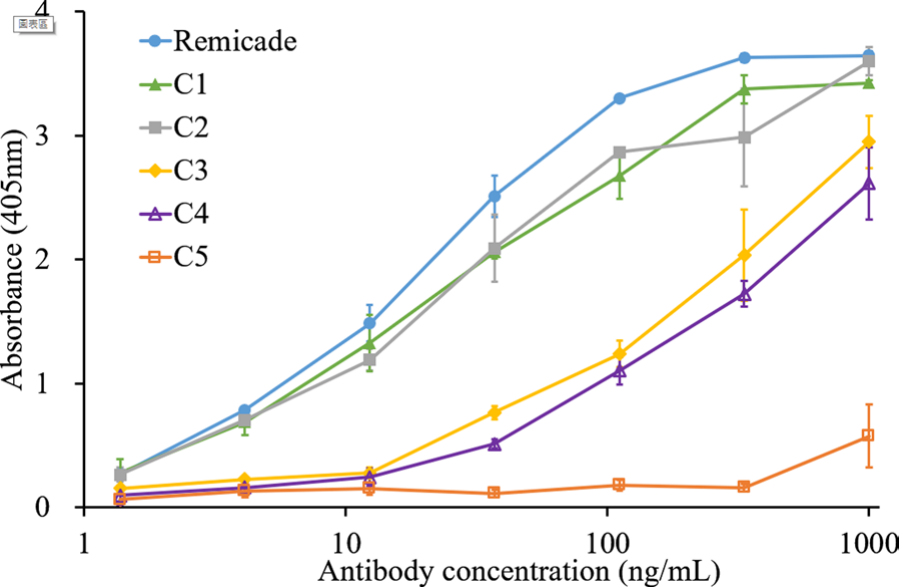
Evaluation of the binding abilities of the antihuman TNF-α Abs. The binding abilities of Remicade (blue ●), C1 (green ▲), C2 (grey ■), C3 (yellow ◆), C4 (purple ○), and C5 (blue □) were examined by performing a TNF-α-based ELISA. The values represent the mean ± SD, *P < 0.0001. Error bar: standard errors of experiments performed in triplicate (https://www.aatbio.com/tools/ec50-calculator)

To confirm that the designed humanized variants have a high binding affinity and whether the Ag-binding abilities of C1–C5 mAbs agree with in silico predictions, we purified the recombinant Remicade and C1–C5 mAbs. The mAbs were added to wells coated with recombinant human TNF-α. The wells were blocked and washed. Then, the horseradish-peroxidase-conjugated goat antihuman IgG-Fc Ab was used to detect the binding between the mAbs and TNF-α at 405 nm. Figure 3 presents ELISA results. The EC50 values were 19.03, 25.83, 26.08, 513.65, and 545.70 ng/mL for the humanized anti-TNF-α mAb variants C1–C5, respectively, and > 1000 ng/mL for the Remicade (Table 1). The R^2^ value of the correlation between the log(EC50) and wRMSD_i_ was 0.901 (Fig. 5A), indicating that our wRMSD_i_ estimations were highly correlated with the EC50 values of the humanized anti-TNF-α candidates.

### In vitro binding affinities of Remicade, C1, C3, and C5 mAb candidates examined using the BLItz affinity measurement system

**Fig. 4:**
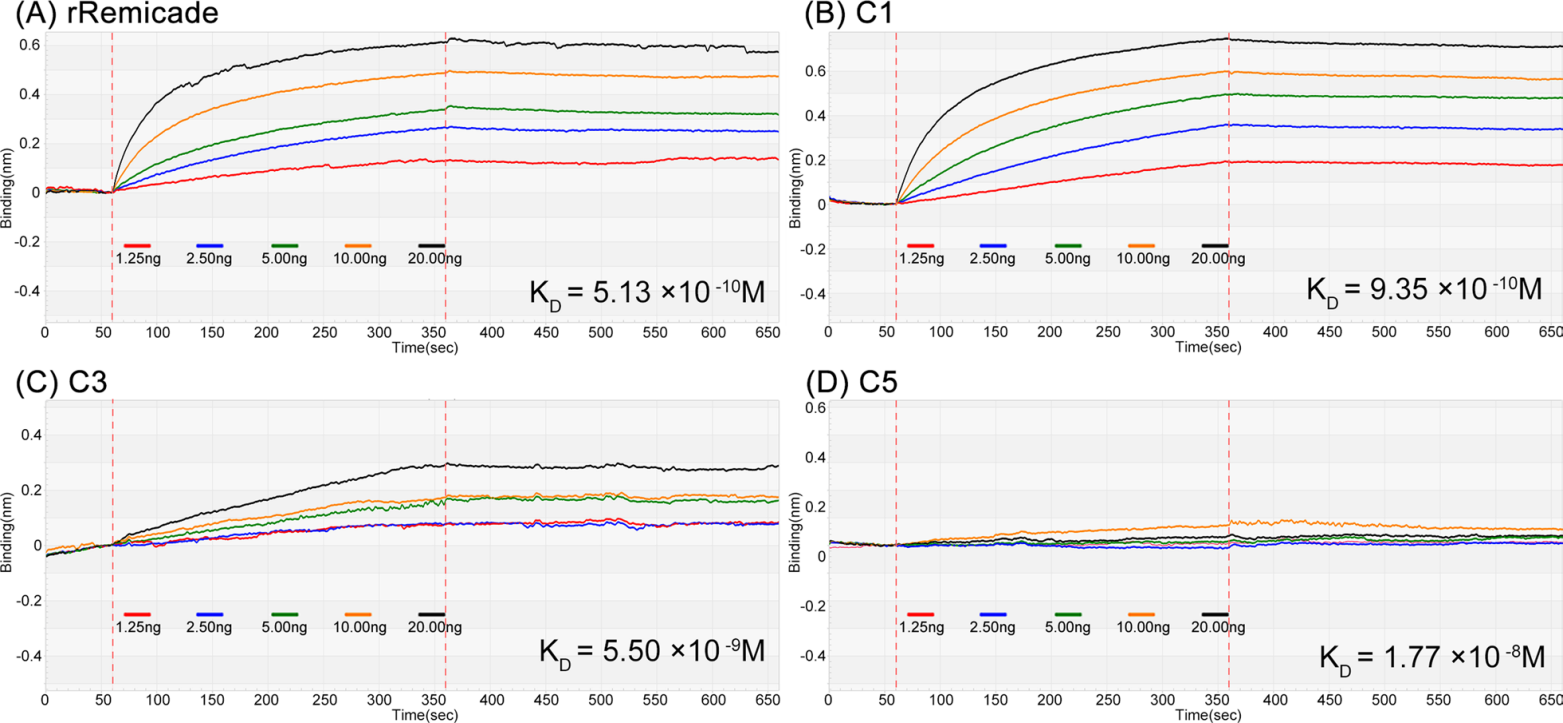
In vitro binding affinities of Remicade, C1, C3, and C5 mAb candidates. The kinetics of the binding of the mAbs to TNF-α were determined through BLI by using human TNF-α–immobilized AR2G biosensors, followed by incubation with different concentrations of anti-TNF-α Abs. The real-time binding curves shown as color lines indicate the global fit determined using black BLItz Pro 1.2 software

**Fig. 5:**
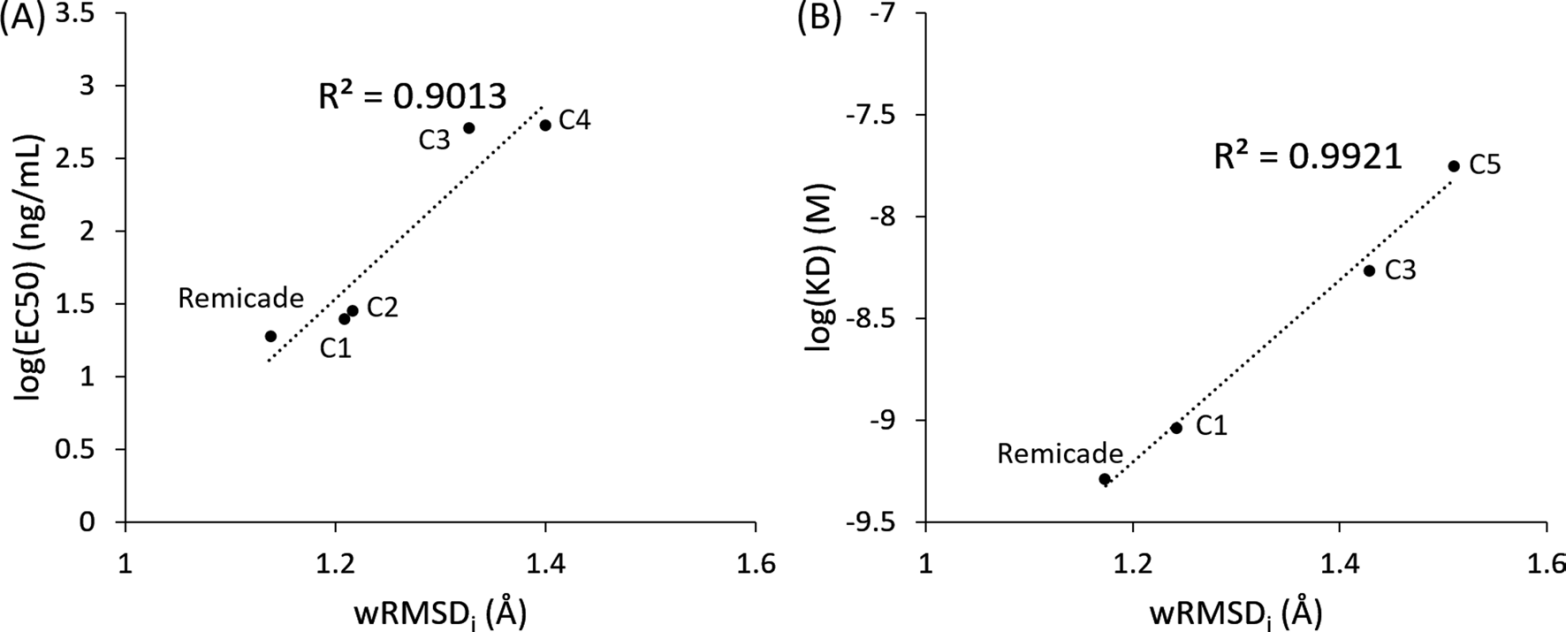
Correlation of the wRMSD_i_ and in vitro binding abilities of Remicade and anti-TNF-α humanized candidates. The in silico simulated wRMSD_i_ was linearly correlated with the logarithms of **A** the EC50 value of anti-TNF-α humanized candidates determined using ELISA (R^2^ = 0.9013) and **B** the K_D_ anti-TNF-α humanized candidates determined using the BLItz affinity measurement system (R^2^ = 0.9921)

To confirm the accuracy of our in silico estimations, we examined the C1, C3, and C5 mAb candidates by using the biolayer interferometry (BLI; BLItz) affinity measurement system because no significant differences were observed between C1 and C2 and between C3 and C4. Human TNF-α was immobilized on amine-reactive second-generation (AR2G) biosensors. Subsequently, the biosensors were incubated with various concentrations of the antihuman TNF-α mAbs (Remicade, C1, C3, and C5). The real-time binding graphs are presented in Fig. 4. The binding ability of the antihuman TNF-α Ab C1 (Fig. 4B) was similar to that of the parent Ab (Remicade; Fig. 4A). The real-time binding curves and kinetics parameters were generated using BLItz Pro 1.2 software. The detected in vitro binding affinities (K_D_) of the Remicade, C1, C3, and C5 mAbs were 5.13 × 10^−10^, 9.35 × 10^−10^, 5.50 × 10^−9^, and 1.77 × 10^−8^ M, respectively (Table 2 and Fig. 4). Our wRMSD_i_ estimations were highly correlated with experimental binding affinities, as indicated by the obtained R^2^ value of 0.9921 (Fig. 5B). The results revealed that our in silico V(D)J recombination mAb platform can yield satisfactory predictions and thus be used to design high-affinity humanized mAbs.

**Table 2:** In vitro binding affinities of Remicade, C1, C3, and C5 mAb candidates

Candidates (mAbs)	wRMSD(Å)	BLItz affinity measurement system		
		Ka (1/Ms)	Kd(1/S)	K_D_ (M)
C1	1.207	1.43 × 10^5^	1.34 × 10^–4^	9.35 × 10^–10^
C3	1.327	2.72 × 10^4^	1.50 × 10^–4^	5.50 × 10^–9^
C5	1.469	2.68 × 10^4^	4.75 × 10^–4^	1.77 × 10^–8^
rRemicade	1.137	1.86 × 10^5^	9.53 × 10^–5^	5.13 × 10^–10^

### Examination of in silico simulation and estimation with anti-EGFR a4.6.1, anti-GPC3 YP9.1, and anti-α4β1 integrin HP1/2L mAbs

To examine whether our in silico method can be used to predict the binding affinity of other mAbs, we used our method to test the three humanized mAbs published in other studies: anti-EGFR a4.6.1 [29], anti-GPC3 YP9.1 [30] and anti-α4β1 integrin HP1/2L [31]. We used our in silico estimation method to derive the wRMSD_i_ of the humanized variants of a4.6.1, YP9.1, and HP1/2L. The EC50 values of the fab8, fab10, fab11, and fab12 of the anti-EGFR a4.6.1 mAb were 0.470, 0.314, 0.162, and 0.078 nM, respectively, whereas our simulated wRMSD_i_ were 3.140, 3.118, 3.066, and 2.997, respectively (Additional file 1: Table S1). The R^2^ value of the correlation between log(EC50) and wRMSD_i_ was 0.9908 (Additional file 1: Fig. S1). The EC50 values of YP9,1, hYP9.1a, and hYP9.1b were 1.8, 47.0, and 6.7 nM, respectively, for the anti-GPC3 YP9.1 mAb (Additional file 1: Table S2), whereas our simulated wRMSD_i_ were 1.995, 2.239, and 2.092, respectively (Additional file 1: Table S2). The R^2^ value of the correlation between log(EC50) and wRMSD_i_ was 0.9999 (Additional file 1: Fig. S2). The EC50 values of HP1/2L, #143, #144, #152, and #208 of the anti-α4β1 integrin HP1/2L mAB were 0.015, 0.53, 0.038, 0.023, and 0.3 nM, respectively, whereas our simulated wRMSD_i_ were 2.116, 3.080, 2.508, 2.484, and 2.787, respectively (Additional file 1: Table S3). The R^2^ value of the correlation between log(EC50) and wRMSD_i_ was 0.8907 (Additional file 1: Fig. S3). These results demonstrated that the predictions of our in silico V(D)J recombination platform were in good agreement with the experimental results, indicating the applicability of our platform (Additional file 1).

## Discussion

We successfully demonstrated that our in silico V(D)J recombination platform can be used to design functional humanized mAbs and accurately predict the affinities of humanized candidates. The results revealed that the wRMSD_i_ was correlated with the logarithm of the binding affinities of the anti-TNF-α mAbs (log(K_D_)) with an R^2^ value of 0.9921. Moreover, after the germline V(D)J recombinant humanization of the Abs, we observed that the platform could effectively convert any nonhuman mAb into low-immunogenicity and high-affinity therapeutic humanized mAbs. The in silico estimation method could not only accurately predict the affinity of the humanized mAbs but also be integrated with other grafting and back mutation methods to predict the optimal humanization conditions of unsolved mouse mAbs. Therefore, our method can facilitate the discovery and development of therapeutic humanized mAbs.

Several essential properties are necessary for the clinical applications of mAbs: high Ag binding activity, high stability, and low immunogenicity [33]. Because human germline genes have less intraclonal somatic hypermutations, humanized Abs with germline gene frameworks might have lower immunogenicity than do humanized mAbs with IgG frameworks [13]. Our in silico V(D)J recombination platform used the germline humanization method to generate low-immunogenicity humanized mAbs. A study successfully used transgenic mice carrying the human Ig gene to develop mAbs with low immunogenicity [34]. For example, XenoMouse was genetically modified by replacing the human Ig gene light chain (κ and λ) and heavy chain loci in endogenous Ig genes, thus enabling the mice to synthesize fully human mAbs upon immunization. For example, panitumumab (Vectibix), a fully human Ab directed against EGFR, is used to treat advanced colorectal cancer [35]. Durvalumab (Imfinzi), a fully human Ab directed against programmed death ligand 1, is used to treat bladder and lung cancer [36]. However, XenoMouse could not convert potential mouse mAbs into potential therapeutic Abs. Human mAbs prepared using XenoMouse must be reimmunized and rescreened each time, and they might have weaker affinities than do mouse-immunized mAbs. Moreover, the XenoMouse technique is expensive. Our in silico V(D)J recombination platform can be used to convert mouse mAbs obtained from conventional mouse experiments into germline-based therapeutic humanized mAbs with high Ag binding activity and stability. The computer-based method can accelerate the humanization process of mouse mAbs and increase their clinical potential.

MD simulation is a robust method for investigating the structures and motions of mAbs. Although web-based homology modeling tools (ABodyBuilder and Kotai Antibody Builder) [37, 38] can be used to predict the three-dimensional mAb structure, matching the CDR loops of mouse mAbs to any known template can be difficult and result in low-quality modeled CDR loops, especially for CDR-H3 [17–20] CDR-H3 is central in Ag binding and contains highly diverse lengths, sequences, and structures because of V(D)J recombination. Liedl et al. subjected five humanized mAbs to MD simulation and found that the conformational diversity of CDR-H3 decreased if it was grafted on the human framework. The inefficient reproduction of the functional conformation of CDR-H3 in humanized candidates can reduce their binding affinity. Therefore, the conformation of CDR-H3 should be described as a structural ensemble rather than a specific structure [39]. In our platform, the motions of CDR loops were recorded as MD trajectories. The diversity of the CDR-H3 of the humanized candidates and mouse mAbs was statistically compared between trajectories. Therefore, the application of MD simulation can maintain the structural ensemble, thus increasing the accuracy of the computational prediction of mAb humanization.

RMSD_i_ is commonly used to compare the sets of MD trajectories [24–27]. If a humanized candidate has a smaller RMSD_i_ in the CDR than the mouse mAb does, then the binding affinity of the candidate to Ags would be similar to that of the mouse Ab [40–42]. However, not every CDR residue is crucial for Ag binding. Padlan et al. examined Ab–Ag interfaces and found that only 21%–28% of CDR residues are used in Ag binding. Weights can be applied to the RMSD method to compare the structure with the binding region. If a residue is more accessible to the solvent, then it might be involved in Ag binding [43]. Accordingly, we added accessibility weights to each CDR residue on the basis of their SASA. Thus, our method could compare binding residues. In addition, Damm et al. described a Gaussian-weighted RMSD method for comparing flexible proteins; the weights of amino acids were undermeasured in the flexible regions of proteins to prevent uncertainty [28]. Some CDR-H3 loops are flexible and have highly diverse shapes [44]. This flexibility can cause uncertainty in the comparison of humanized candidates with these mAbs. In MD simulations, high vibrational residues present high motional standard deviations. Therefore, we added weights that were inversely proportional to the simulational motional standard deviation of each CDR residue to prevent the effects of CDR loop flexibility. These two weights increased the accuracy of our in silico estimation method. In the anti-TNF-α mAbs, CDR-H3 was not particularly flexible; however, wRMSD_i_ could improve R^2^ from 0.9855 to 0.9921. In the humanized a4.6.1 containing highly flexible CDR-H3 (Additional file 1), the R^2^ values of the best four variants improved from 0.952 to 0.990. In the humanized HP1/2L (Additional file 1), the weighting applied in more flexible CDR-H3 improved the R^2^ value from 0.712 to 0.850. The R^2^ differences between the unweighted and weighted humanized mAbs are listed in Additional file 1: Table S4, indicating that wRMSD_i_ could improve the prediction of the binding affinities of the humanized candidates.

## Conclusion

We developed an in silico V(D)J recombination platform that combines germline humanization to prevent HAMA immunogenicity and can predict the best humanized mAb candidates. We designed five germline-humanized anti-TNF-α variants. The variants were subsequently subjected to MD simulations, and their structural similarities were estimated by comparing them with the original mouse mAb. Furthermore, we used wRMSD_i_ to adjust the effect of each CDR residue on the basis of the SASA and motional standard deviation to compare crucial CDR residues and prevent the effects of highly flexible CDR loops. The predictions of wRMSD_i_ agreed with our experimental results, and the R^2^ value of the correlation between wRMSD_i_ and log(K_D_) was 0.992. In addition, the binding affinity of the best humanized candidate C1 was similar to that of the chimeric parental mAb. Moreover, we tested our in silico estimation method by using the anti-EGFR a4.6.1, anti-GPC3 YP9.1, and anti-α4β1 integrin HP1/2L mAbs. The R^2^ values of the correlation of wRMSD_i_ with the log(EC50) of the humanized a4.6.1, YP9.1, and HP1/2L mAbs were 0.991, 1.000, and 0.891, respectively, indicating that our platform can be universally used to predict humanized mAb candidates. Overall, our in silico V(D)J recombination platform can be used to convert mouse mAbs into low-immunogenicity and high-affinity therapeutic humanized mAbs and thus facilitate the discovery of mAbs. Our in silico estimation method could effectively determine candidates with the highest binding affinities and can be used for Abs with further back mutations. Advancements in computational methods and computing power can address challenges in designing humanized mAbs.

## Methods

### Equations of wRMSD_i_

To prevent sampling bias, murine and humanized Ab models were built using a crystal structure or through homology modeling and then equilibrated and simulated in 16 parallel sets of 100-ns MD simulations. The resulting simulation trajectories were analyzed, and wRMSD_i_ was calculated by comparing interatomic distances between the Cα atoms of CDR residues. The RMSD method was used to quantitatively determine interatomic distances between CDR Cα atoms. A distance matrix with all CDR Cα–Cα distances was recorded in each snapshot, and the matrices of each humanized candidate were compared with those of the original murine Ab to derive the RMSD_i_ of each CDR residue. The RMSD_i_ was then reweighted depending on the accessibility and movability of the wRMSDi. Relevant equations are listed below:

$${\mathrm{RMSD}}_{i}=\sqrt{\frac{\sum_{j}{\left({d}_{ij}-{d}_{ij}^{0}\right)}^{2}}{n}}$$

1 $${\mathrm{wRMSD}}_{i}={\sum }_{i}\frac{{w}_{i-accessibility }{w}_{i-movability}{RMSD}_{i}}{N}$$

Here, *RMSD*_*i*_ is the interatomic RMSD of the Cα atom of the *i*th CDR residue, *d*_*ij*_ is the distance between the *i*th and *j*th Cα atom, *d*_*ij*_^*0*^ is the corresponding distance in the snapshots of murine simulations, *n* is the number of interatomic interactions, and *N* is the number of CDR residues. The weights *w*_*i-accessibility*_ and *w*_*i-movability*_ are determined using the following equations.

$${w}_{i-accessibility}=1/\left(\frac{{S}_{i}}{{S}_{i.avg}}\right)$$

2 $${w}_{i-movability}=1/\left(\frac{{RMSD}_{i}^{0}}{{RMSD}_{i.avg}^{0}}\right)$$

The w_i-accessibility_ is the weight determined by measuring the SASA of each CDR residue (*S*_*i*_). The CDR residues with more solvent accessible areas were more accessible during Ag binding. w_i-movability_ is based on the motional standard deviation of the *i*th residue from the *RMSD*^*0*^_*i*_, which is the standard deviation of the *i*th CDR residue in the original murine mAb MD simulation. A higher *RMSD*^*0*^_*i*_ indicates that a residue is more flexible; thus, its weight should be decreased. In addition, *S*_*i.avg*_ and *RMSD*^*0*^_*i.avg*_ are fractional denominators used to maintain the relative value of *wRMSD*_*i*_.

### Simulation environments of humanized mAb candidates

All the models of humanized mAb candidates were built using ABodyBuilder web services [37], and the three-dimensional Remicade model was obtained from the Protein Data Bank database (PDB ID: 4G3Y) [45]. Subsequently, the murine Abs and humanized candidates were simulated using 16 separate replicas of simulations to obtain adequate samples (in terms of entropy) [46] by using the AMBER CUDA-accelerated PMEMD program [47]. Each replica began with a 10 000-step minimization, followed by 1-ns heating, 40-ns equilibrium, and 80-ns production steps. The time step of 2 fs used the NVT canonical ensemble with Langevin dynamics, and the temperature was set at 310 K. The GB/SA solvation condition (igb = 5) [48] was used, and SHAKE bond restraints were used to constraint hydrogen-involving bonds after energy minimization. In each 80-ns production simulation, the distance between the Cα atoms of CDRs was recorded to compare motional differences between murine and humanized mAbs for calculating the RMSD_i_. In addition, each MD snapshot was used to calculate the SASA of each CDR residue by using the freesasa program [49].

### Preparation of recombinant anti-TNF-α Abs

The light and heavy chains of antihuman TNF-α Abs were cloned into a pLNCX vector. In this experiment, 30 μg of the antihuman TNF-α Ab plasmid was transfected into 6 × 10^7^ Expi293 cells by using Expifectamine reagent. The media were harvested for subsequent experiments 5 days after transfection. The antihuman TNF-α Abs were purified through protein A sepharose fast flow chromatography (GE Healthcare, Little Chalfont, UK). The Ab concentration was determined using the bicinchoninic acid assay (BCA) method, and the purity of purified antihuman TNF-α Abs was analyzed through sodium dodecyl sulfate–polyacrylamide gel electrophoresis in reducing conditions.

### Analysis of the binding affinity of antihuman TNF-α Abs

Multiwell plates were coated with 50 ng/well of recombinant human TNF-α. The plates were blocked by incubation with 5% skimmed milk/phosphate-buffered saline (PBS) for 2 h at 37 °C and subsequently washed twice in 0.05% Tween-20/PBS. Graded concentrations of the recombinant humanized Ab or mAb were diluted with PBS containing 2% skimmed milk and added to the wells (50 μL/well) at room temperature for 1 h. Anti-TNF-α Abs bound to TNF-α were detected by adding the horseradish-peroxidase-conjugated goat antihuman IgG-Fc Ab (0.5 μg/mL) at room temperature for 1 h. The plates were washed with PBST three times and PBS once, and the bound Ab was measured by adding 150 μL/well of 2-azinobis (3-ethylbenzthiazoline-6-sulfonic acid) (ABTS) solution, 0.003% H_2_O_2_, and 100 mM phosphate–citrate (pH = 4.0) for 30 min at room temperature. Absorbance was measured at 405 nm through blank subtraction. All experiments were performed in triplicate, and data are presented as the mean ± standard deviation.

### Analysis of the binding affinity of humanized anti-TNF-α Abs by using the BLItz affinity measurement system

Human TNF-α was immobilized on the AR2G biosensor according to the manufacturer’s instructions. The sensor surface was activated with 20 mM N-(3-dimethylaminopropyl)-N′-ethylcarbodiimide (EDC) and 10 mM s-NHS at room temperature. Next, human TNF-α (5 μg/mL in 10 mM acetate buffer, pH 5) was immobilized on the surface. Any remaining activated groups were quenched using 1 M ethanolamine (pH 8.5). To examine whether the immobilized human TNF-α could specifically bind to the anti-TNF-α Ab, the sensors were subsequently incubated with various concentrations of the anti-TNF-α Ab at room temperature. The real-time binding curves and kinetic parameters were generated using black BLItz Pro 1.2 software (ForteBio, Fremont, CA).

## Materials or reagents

### Drug and reagents

We purchased ABTS solution from Sigma-Aldrich (St. Louis, MO, USA). AR2G biosensors were purchased from Fortebio (San Jose, CA, USA). EDC was purchased from Aladdin Chemistry Co. (Shanghai, China). Recombinant human TNF-α was obtained from R&D Systems (Minneapolis, MN, USA).

### Cells

293 T cells were grown in Dulbecco’s modified Eagle’s medium (Sigma-Aldrich) containing 10% bovine calf serum (HyClone) and 100 U/mL penicillin and streptomycin (Invitrogen, Calsbad, CA) at 37 °C in a humidified atmosphere of 5% CO_2_. Expi293 cells were grown in the Expi293 expression medium (Gibco Laboratories, Grand Island, NY, USA) and cultured in shaker flasks at 120 rpm and 37 °C in an incubator with 8% CO_2_.

## Supplementary Information

**Additional file 1. Supplementary information: Table S1.** Calculated wRMSD_i_ values of the anti-EGFR a4.6.1 mAb and experimental results. **Figure S1.** Correlation of wRMSD_i_ with the log(EC50) of the anti-EGFR a4.6.1 mAb. **Table S2.** Calculated wRMSD_i_ values of the anti-GPC3 YP9.1 scFv and experimental results. **Figure S2.** Correlation of wRMSD_i_ with the log(EC50) of the anti-GPC3 YP9.1 scFv. **Table S3.** Calculated wRMSD_i_ values and experimental results of the humanized anti-α4β1 integrin variants. **Figure S3.** Correlation of wRMSD_i_ with the log(EC50) of humanized anti-α4β1 integrin variants. **Table S4.** Correlation between RMSDi and log(EC50) or log(K_D_) with and without weighting adjustments.

## Data Availability

All data generated or analyzed during this study are included in this published article and its Additional files.
