# Supplementary material for: A universal in silico V(D)J recombination strategy for developing humanized monoclonal antibodies
Source: J Nanobiotechnology. 2022 Jan 31;20:58. doi: 10.1186/s12951-022-01259-2 (PMC8805405; doi:10.1186/s12951-022-01259-2)
Supplement: Supplementary file 1 — Additional file 1. Supplementary information: Table S1. Calculated wRMSDi values of the anti-EGFR a4.6.1 mAb and experimental results. Figure S1. Correlation of wRMSDi with the log(EC50) of the anti-EGFR a4.6.1 mAb. Table S2. Calculated wRMSDi values of the anti-GPC3 YP9.1 scFv and experimental results. Figure S2. Correlation of wRMSDi with the log(EC50) of the anti-GPC3 YP9.1 scFv. Table S3. Calculated wRMSDi values and experimental results of the humanized anti-α4β1 integrin variants. Figure S3. Correlation of wRMSDi with the log(EC50) of humanized anti-α4β1 integrin variants. Table S4. Correlation between RMSDi and log(EC50) or log(KD) with and without weighting adjustments. [file 12951_2022_1259_MOESM1_ESM.pdf]

A universal in silico V(D)J recombination strategy for developing humanized monoclonal antibodies

Yuan-Chin Hsieh[1,6#], Jun min Liao[1#], Kuo-Hsiang Chuang[2, 3], Kai-Wen Ho[4], Shih-Ting Hong[4], Hui-Ju Liu[4], Bo-Cheng Huang[5], I-Ju Chen[7], Yen-Ling Liu[8], Jaw-Yuan Wang[1,4,9,10,11,12], Hsiang-Lin Tsai[9,10], Yu-Cheng Su[13], Yen-Tseng Wang[1,14*], Tian-Lu Cheng[1,8*]

[1]Center for Biomarkers and Biotech Drugs, Kaohsiung Medical University, 100 Shih-Chuan First Road, Kaohsiung 80708, Taiwan

[2]Graduate Institute of Pharmacognosy, Taipei Medical University, 250 Wuxing Street, Taipei 11031, Taiwan

[3]Ph.D. Program for Clinical Drug Discovery from Botanical Herbs, Taipei Medical University, 250 Wuxing Street, Taipei 11031, Taiwan

[4]Graduate Institute of Medicine, College of Medicine, Kaohsiung Medical University, Kaohsiung, Taiwan

[5]Institute of Biomedical Sciences National Sun Yat-Sen University, Kaohsiung, Taiwan

[6]School of Medicine for International Students, I-Shou University, Kaohsiung, Taiwan

[7]School of Medicine, I-Shou University, Kaohsiung, Taiwan

[8]Department of Biomedical Science and Environmental Biology, Kaohsiung Medical University, 100 Shih-Chuan First Road, Kaohsiung 80708, Taiwan

[9]Division of Colorectal Surgery, Department of Surgery, Kaohsiung Medical University Hospital, Kaohsiung Medical University, Kaohsiung, Taiwan

[10]Department of Surgery, Faculty of Medicine, Kaohsiung Medical University, Kaohsiung, Taiwan

[11]Graduate Institute of Clinical Medicine, College of Medicine, Kaohsiung Medical University, Kaohsiung 80756, Taiwan

[12]Center for Cancer Research, Kaohsiung Medical University, Kaohsiung 80708, Taiwan

[13]Department of Biological Science and Technology, National Yang Ming Chiao Tung University, Hsin-Chu 300, Taiwan

[14]School of Post-Baccalaureate Medicine, College of Medicine, Kaohsiung Medical University, 100 Shih-Chuan First Road, Kaohsiung 80708, Taiwan

Supplementary information

Availability of referenced humanization experiments

Although many humanized Abs have been developed, the availability of data regarding failed cases is limited. Most of the studies have reported only successful cases. Furthermore, mutations are typically present in CDRs rather than in framework regions. Although some studies have reported failed attempts, they did not clearly indicate the binding affinity (KD) or EC50 of the Abs. To validate our in silico method, we searched for studies providing a list of humanized Abs and their corresponding binding properties and chose the humanized anti-epidermal growth factor receptor (EGFR) [1], anti-glypican-3 (GPC3) [2], and anti-α4β1 integrin [3] mAbs to prove our in silico estimation method.

Application of our in silico method to the anti-EGFR a4.6.1 mAb

To validate our computational strategy, we tested our in silico simulation method for the anti-EGFR a4.6.1 mAb [1]. The results of the best four candidates are listed in **Table S1**. The $wRMSD_i$ values of fab12, fab11, fab10, and fab8 were 2.997, 3.066, 3.118, and 3.140, respectively. Furthermore, the $wRMSD_i$ values of a4.6.1 variants were high (>2.5Å), indicating that the CDR of a4.6.1 was flexible. The R^2 value of the correlation between the logarithm of EC50 and $wRMSD_i$ was 0.9908 (**Fig. S1**). This finding indicated that our in silico prediction method can be used to estimate the binding affinities of humanized candidates, especially those with low $wRMSD_i$ values. If the structural difference between the humanized candidate and the original murine mAb is large, its effect on binding affinity would be less predictable.

Table S1. Calculated $wRMSD_i$ values of the anti-EGFR a4.6.1 mAb and experimental results.

Candidates	$wRMSD_i$	EC50 (nM, reference)	Simulation ranking	Experiment ranking
fab12	2.997	0.0784	1	1
fab11	3.066	0.1617	2	2
fab10	3.118	0.3136	3	3
fab8	3.140	0.4704	4	4

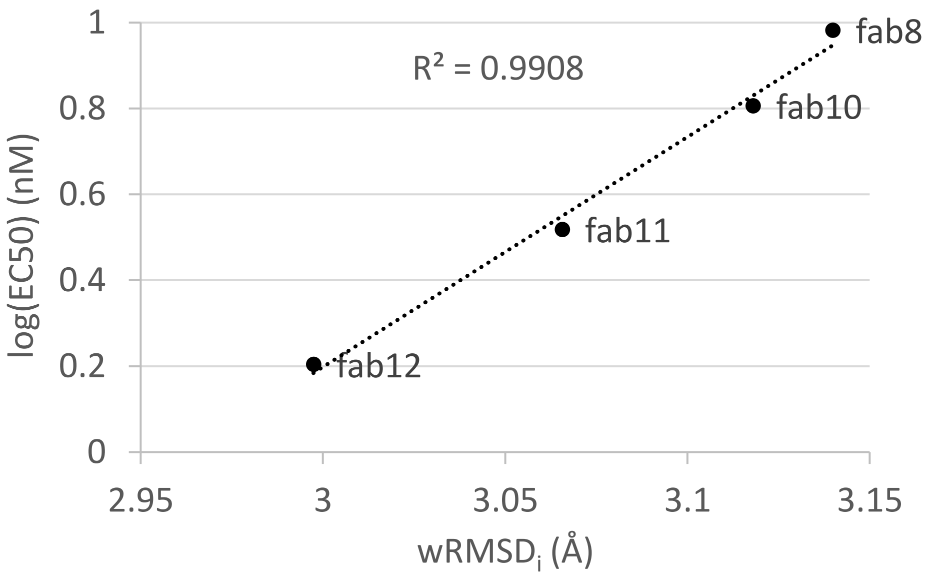

Figure S1. Correlation of $wRMSD_i$ with the log(EC50) of the anti-EGFR a4.6.1 mAb. In the best four humanized candidates, the R^2 value of 0.9908 indicated that $wRMSD_i$ was highly correlated with the logarithm of EC50.

Application of our in silico method to the anti-GPC3 single-chain (scFv) Ab

We tested our in silico simulation method for the humanized anti-GPC3 scFv mAb [2]. The original mouse mAb YP9.1 was used as the structural reference for comparisons. The EC50 and wRMSD$_i$ results are listed in **Table S2**. The wRMSD$_i$ values of YP9.1, hYP9.1a, and hYP9.1b were 1.995, 2.239, and 2.092, respectively. The R^2 value of the correlation between log(EC50) and wRMSD$_i$ was 0.9999 (**Fig. S2**).

Table S2. Calculated wRMSD$_i$ values of the anti-GPC3 YP9.1 scFv and experimental results.

Candidates	wRMSD$_i$ (Å)	EC50 (nM, reference)	Simulation ranking	Experiment ranking
YP9.1	1.995	1.8	1	1
hYP9.1a	2.239	47	3	3
hYP9.1b	2.092	6.7	2	2

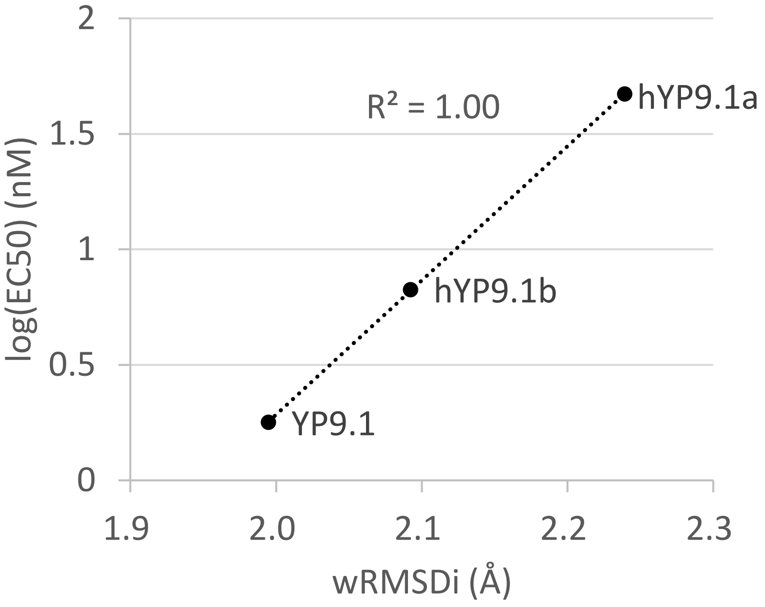

Figure S2. Correlation of wRMSD$_i$ with the log(EC50) of the anti-GPC3 YP9.1 scFv. The R^2 value of 0.9999 indicated that wRMSD$_i$ was highly correlated with the logarithm of EC50 in humanized anti-GPC3 variants.

Application of our in silico method to the anti-α4β1 integrin HP1/2L mAb

We tested our in silico simulation method for the humanized anti-α4β1 integrin mAb [3]. The mouse mAb HP1/2L was set as the structural reference for comparisons. The EC50 and $wRMSD_i$ results are listed in **Table S3**. The R^2 value of the correlation between the logarithm of EC50 and $wRMSD_i$ was 0.8907 (**Fig. S3**).

Table S3. Calculated $wRMSD_i$ values and experimental results of the humanized anti-α4β1 integrin variants.

Candidates	$wRMSD_i$ (Å)	EC50 (nM, reference)	Simulation ranking	Experiment ranking
HP1/2L	2.116	0.015	1	1
#143	3.080	0.53	5	5
#144	2.508	0.038	3	3
#152	2.484	0.023	2	2
#208	2.787	0.30	4	4

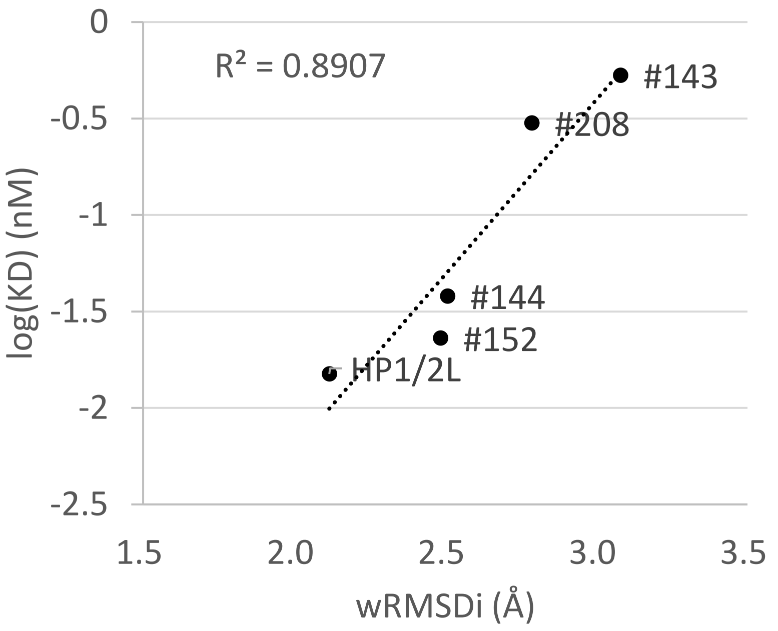

Figure S3. Correlation of $wRMSD_i$ with the log(EC50) of humanized anti-α4β1 integrin variants. The R^2 value of 0.8907 indicated that $wRMSD_i$ was highly correlated with the logarithm of EC50 in the humanized anti-α4β1 integrin variants.

Weighted and unweighted $RMSD_i$

We used the SASA and motion fluctuations as weights to adjust RMSDi values. The weighted $RMSD_i$ was more correlated with the logarithm of EC50 or K_D compared with the unweighted $RMSD_i$ (**Table S4**). The results indicated that weighting improved the prediction of the binding affinity of the humanized candidates.

Table S4. Correlation between $RMSD_i$ and log(EC50) or log(K_D) with and without weighting adjustments.

Humanized Antibody	R^2 (Å) unweighted	R^2 (Å) weighted	Improved R^2
**anti-TNFα_EC50**	0.9010	0.9013	0.0003
**anti-TNFα_K_D**	0.9855	0.9921	0.0066
**anti-EGFR a4.6.1**	0.9521	0.9908	0.0387
**anti-GPC3 YP9.1**	0.9606	0.9724	0.0118
**anti-α4β1 integrin HP1/2L**	0.7604	0.8907	0.1303

Reference

1. Presta LG, Chen H, O'connor SJ, Chisholm V, Meng YG, Krummen L, Winkler M, Ferrara N: **Humanization of an anti-vascular endothelial growth factor monoclonal antibody for the therapy of solid tumors and other disorders.** *Cancer research* 1997, **57:**4593-4599.
2. Zhang Y-F, Ho M: **Humanization of high-affinity antibodies targeting glypican-3 in hepatocellular carcinoma.** *Scientific reports* 2016, **6:**1-11.
3. Hanf KJ, Arndt JW, Chen LL, Jarpe M, Boriack-Sjodin PA, Li Y, van Vlijmen HW, Pepinsky RB, Simon KJ, Lugovskoy A: **Antibody humanization by redesign of complementarity-determining region residues proximate to the acceptor framework.** *Methods* 2014, **65:**68-76.
